# Flange Fracture and Dislocation: An Unusual Complication of Percutaneous Tracheostomy

**DOI:** 10.7759/cureus.26426

**Published:** 2022-06-29

**Authors:** Ilya Shnaydman, Jeffrey Baum, Liran Barda, Shrey Modi, Joyce Kaufman, Rishi Rattan

**Affiliations:** 1 Trauma and Critical Care, Department of Surgery, Westchester Medical Center, New York, USA; 2 Surgery, Mount Sinai South Nassau, Oceanside, USA; 3 Trauma and Acute Care Surgery, Ryder Trauma Center – Jackson Memorial Hospital, Miami, USA

**Keywords:** case report, fracture, flange, airway loss, tracheostomy, percutaneous

## Abstract

Percutaneous dilational tracheostomy (PDT) is a commonly used technique in intensive care units (ICUs) for persistent respiratory failure. Early complications of placement includeairway loss, bleeding, and tracheal ring fracture. Tracheostomy tube fracture is a rarely reported complication that can lead to loss of airway and require emergent intervention. We present two case reports of tracheostomy flange fracture and dislocation after PDT. Shortly after insertion, the tracheostomy flange was incidentally noted to have irreparably fractured and separated from the outer cannula. Both patients were orotracheally intubated and converted to open revisional surgical tracheostomy. Outer cannula separation from the flange is a rare but important complication of PDT due to the risk of occult airway loss. The tracheostomy tube supplied in the PDT set is manufactured in three parts and the plastic outer cannula is snapped to the silicone flange during manufacturing. The flange is not meant to be separated during insertion or use. PDT insertion requires significant force to be exerted, as the catheter has to travel through the subcutaneous tissue of the neck before entering the trachea. These cases suggest that the junction of the flange and the outer cannula may be a weak point and that fracture and dislocation at that site may occur due to excessive or misdirected force. Dislocation may cause loss of airway and a need for orotracheal intubation as performed in our cases. Understanding this complication and carefully examining the flange after placement is essential for early recognition to avoid loss of airway.

## Introduction

Percutaneous dilational tracheostomy (PDT) by the Seldinger technique has become a commonly used method for tracheostomy and has overtaken open surgical tracheostomy (OST) in most ICUs [1]. Fracture of a tracheostomy tube is a rare complication of tracheostomy. It can present as an asymptomatic breakage, a foreign body in the tracheobronchial tree that may lead to aspiration pneumonia, or an airway emergency [2-6]. Prior reports of tube fractures were attributed to prolonged use, improper care, and inability to perform timely maintenance for routine wear and tear [5-8]. Most fractures occurred ≥ 1 year (range 1-14 years) after placement. There is only a single published case report of an early fracture and dislocation of the outer cannula from the flange, three days after a PDT, using a Ciaglia Blue Rhino® (Cook Medical, Bloomington, IN) [9].

We report two cases of tracheostomy flange separation within hours of performing a percutaneous tracheostomy using the tracheal cannula supplied in Ciaglia Blue Rhino® G2 advanced percutaneous tracheostomy introducer sets, with the 8F Shiley tracheostomy tube, Part # g53175, C-PITS-100-HC-G-PERC8 with the supplied dilator 28F, and the immediate impact and management of this complication. Over the years, various guidelines have been compiled by competent agencies that advise on the proper techniques for performing a PDT. The two cases happened at different time points nearly one year apart, with two completely different operating teams (an attending, a fellow, a resident, and a respiratory technician). The performing operators in both instances had >500 case experience and followed the guidelines set forth by the American Association of Surgical Trauma (AAST). Individual patient records and images were acquired through a review of electronic medical records, and all patient details were de-identified and fully anonymized. Both patients presented to a large, urban academic medical center between 2018 and 2020.

## Case presentation

Case 1

**Figure 1:**
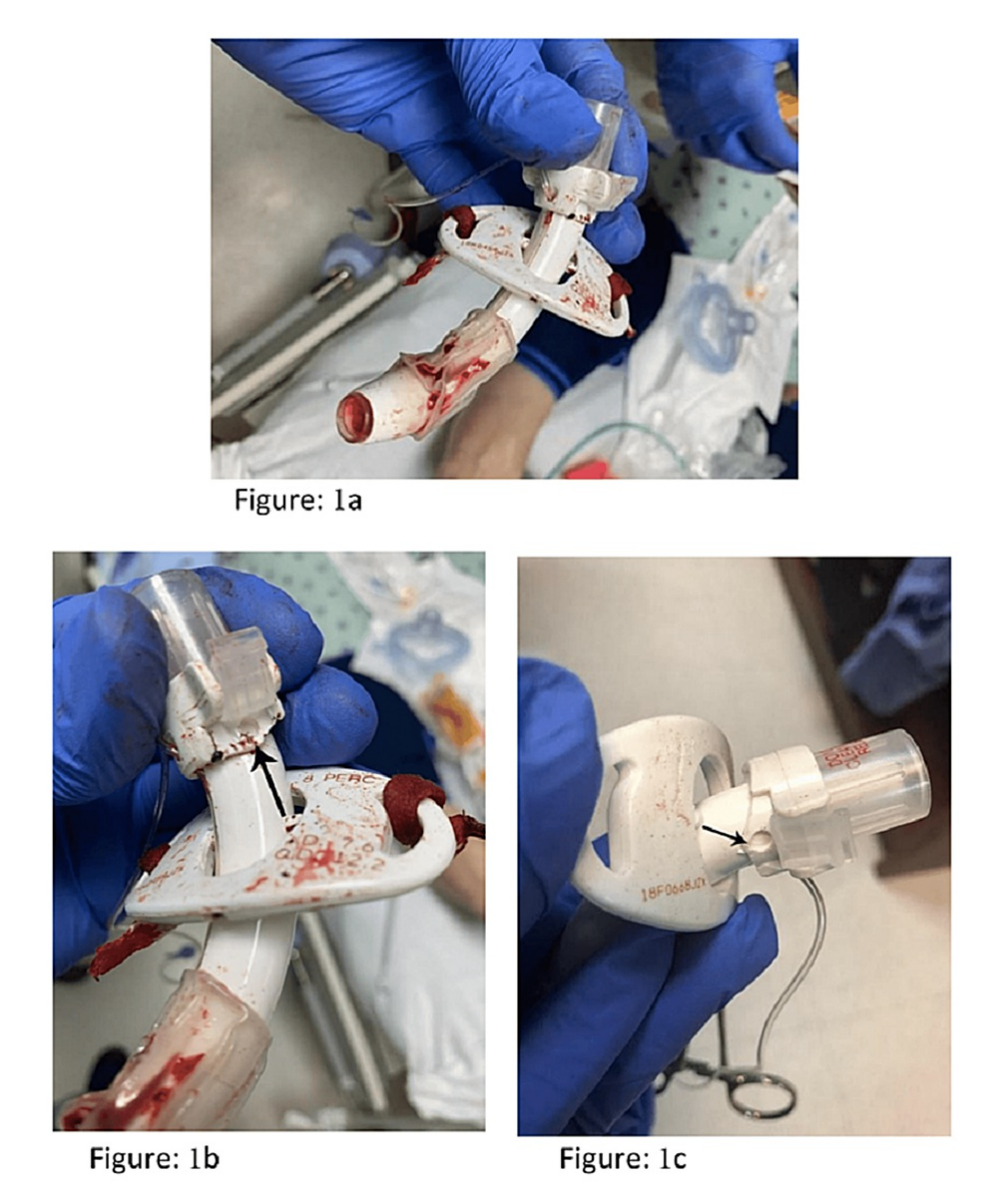
Flange dislocation of percutaneous tracheostomy (1a); another view of the flange dislocation, with an arrow indicating the proper flange pin position (1b); flange dislocation from the second case with an arrow demonstrating the proper flange pin position (1c)

A 53-year-old male with complicated type 1 diabetes mellitus and seizure disorder with reflux pancreatitis through his pancreaticovesicular anastomosis after his kidney-pancreas transplant underwent conversion to a pancreaticoenteric anastomosis. His postoperative course was complicated by an anastomotic leak resulting in respiratory failure and prolonged intubation. The patient was taken for bronchoscopic-guided PDT. Immediately upon completion of the procedure, the flange was noted to have separated from the outer cannula (Figures 1a-1c).

Attempting to reseat the outer cannula into the flange was not successful. To avoid another complication, the decision was made to convert to open tracheostomy and insert a new cannula under direct visualization. The patient was reintubated and returned to the operating room and an open tracheostomy was performed uneventfully.

Case 2

A 74-year-old female whose orthotopic liver transplant was complicated by rejection developed respiratory failure due to disseminated aspergillosis. She underwent a bronchoscopic-guided PDT. Several hours later, the flange was noted to be separated from the cannula and was not able to be reseated. The decision was made to convert to open tracheostomy for placement under direct visualization to avoid any further complications. The patient was orotracheally intubated, taken to the operating room, and underwent an uneventful open tracheostomy.

## Discussion

Percutaneous dilatational tracheostomy over a guidewire was first described by Ciaglia in 1985. The most common indications for PDT in the ICU are to facilitate weaning in prolonged intubated patients, optimize tracheobronchial airway clearance, and minimize sedation [1]. The complications associated with PDT include loss of airway, bleeding, tracheal ring fracture, and device occlusion [1,5-6,10]. Fracturing was more common when tubes were made from metal alloys where manufacturing defects and corrosion played a more important role in failure [11-14].

**Figure 2:**
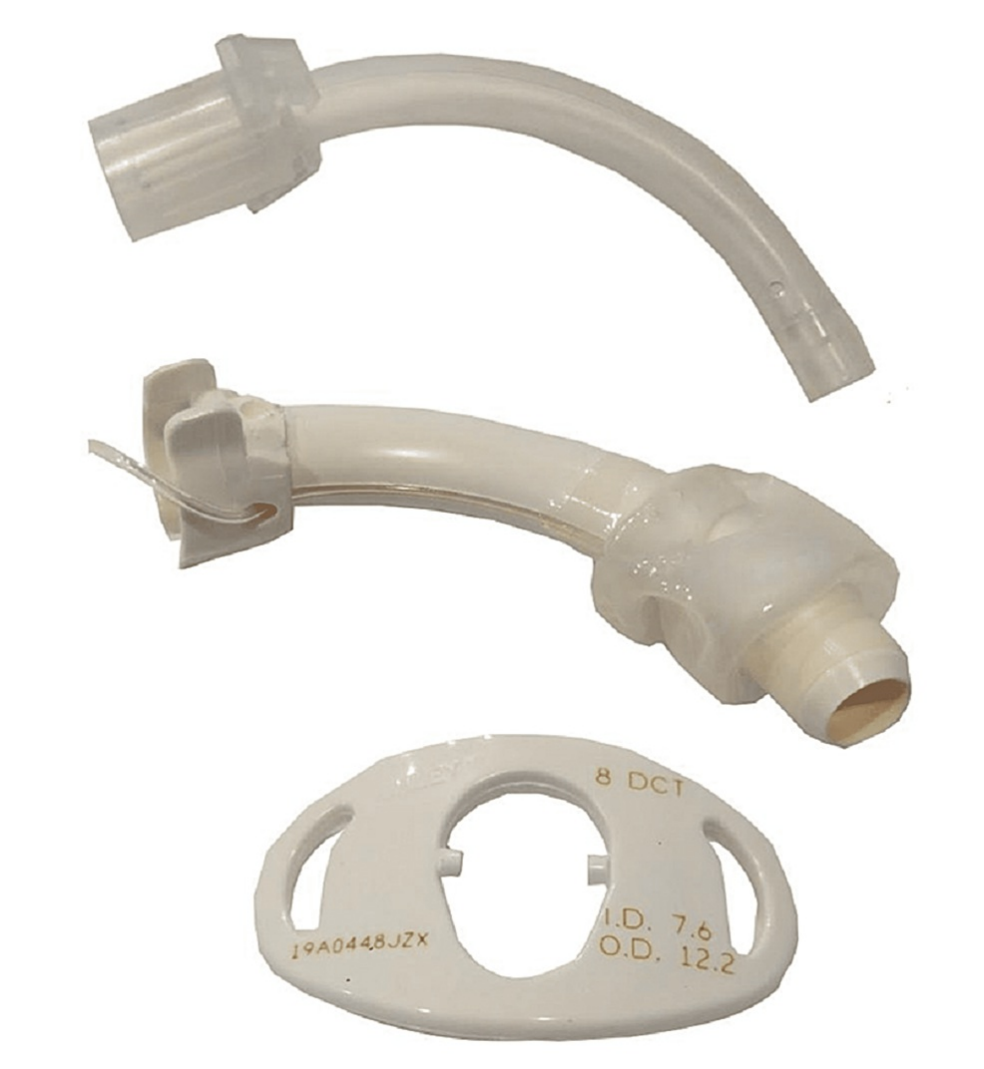
Components of Shiley tracheostomy - inner cannula, outer cannula, and flange (from top to bottom)

With the introduction of polyvinyl chloride (PVC), silicone, and other materials, the incidence of this particular complication has decreased substantially. Percutaneous tracheostomy tubes have a beveled distal tip and a low-profile cuff designed to reduce insertion force [15-16]. The tracheostomy tube is manufactured in three parts (Figure 2).

The inner cannula, which snaps into place inside the outer cannula once the latter is in the airway; the outer cannula; and the silicone flange, which is attached to the outer cannula on a restricted pivot point allowing the tube to be secured to the patient. The flange and outer cannula are snapped together during the manufacturing process and are not meant to be separated during placement or use. Communication from Cook (Bloomington, IN) stated that should such an event occur, the recommendation is to discard the tracheostomy tube and proceed with a new kit. The pivot point allows for a more comfortable seating against the patient’s neck and helps keep the outer cannula centered in the trachea. This allows for proper angulation after placement but creates potential shearing forces that can fracture the connection between the outer cannula and flange, if not accounted for during insertion [9,15]. As the tracheostomy tube is inserted, the tissue is dilated (as opposed to an open tracheostomy where the dissection is first performed to expose the trachea and then the tracheostomy tube is inserted), therefore, the significant force required for a PDT may have contributed to the flange fracture and dislocation. This complication occurred with a commonly used PDT kit but is not limited to this particular model and may affect other kits as well.

We present two case reports that demonstrate a rare hardware complication of percutaneous tracheostomy in which the outer cannula separated from the flange within hours of performing PDT, leading to the impending loss of airway. Both procedures used new tubes placed by different operating teams. On examination, the tracheostomy tubes were cleanly separated at the plastic, pivoting joint between the silicone flange and the PVC outer cannula (Figures 2a-2c).

In both cases, the complication was managed with orotracheal intubation and conversion to open tracheostomy, however, they may have also been rescued by a second attempt at PDT using the existing tracheostomy tract and the Seldinger technique. These two complications were reported to the company and surveillance is being performed to identify if a hardware or production quality issue exists.

## Conclusions

Insertion of a percutaneous tracheostomy requires significant force to be exerted, as the catheter has to travel through the subcutaneous tissue of the neck before entering the trachea, as opposed to an open tracheostomy. Given the mobility of the pivot point, force may be applied in an unintended direction and may lead to fracturing and dislocation of the flange. This particular PDT kit was a commonly used one, but this complication could affect other kits as well. It is imperative for the clinician to understand the construction of the percutaneous flange and the force required to properly seat the tube. Careful examination of the flange after placement is essential for early recognition to avoid the dreaded complication of airway loss. In both cases, the flange was not able to be reseated and the patient had to undergo a second orotracheal intubation and revision surgical open tracheostomy.
